# Genome-wide map of quantified epigenetic changes during *in vitro* chondrogenic differentiation of primary human mesenchymal stem cells

**DOI:** 10.1186/1471-2164-14-105

**Published:** 2013-02-15

**Authors:** Sarah R Herlofsen, Jan Christian Bryne, Torill Høiby, Li Wang, Robbyn Issner, Xiaolan Zhang, Michael J Coyne, Patrick Boyle, Hongcang Gu, Leonardo A Meza-Zepeda, Philippe Collas, Tarjei S Mikkelsen, Jan E Brinchmann

**Affiliations:** 1 Institute of Immunology and Norwegian Center for Stem Cell Research, Oslo University Hospital Rikshospitalet, Oslo 0424, Norway; 2 Department of Tumor Biology, Institute for Cancer Research, Oslo University Hospital Radiumhospitalet, Oslo 0424, Norway; 3 Broad Institute of MIT and Harvard, Massachusetts 02142, USA; 4 Institute of Basic Medical Sciences, Department of Biochemistry, University of Oslo, Oslo 0317, Norway; 5 Harvard Stem Cell Institute and Department of Stem Cell and Regenerative Biology, Harvard University, Cambridge, MA 02138, USA

## Abstract

**Background:**

For safe clinical application of engineered cartilage made from mesenchymal stem cells (MSCs), molecular mechanisms for chondrogenic differentiation must be known in detail. Changes in gene expression and extracellular matrix synthesis have been extensively studied, but the epigenomic modifications underlying these changes have not been described. To this end we performed whole-genome chromatin immunoprecipitation and deep sequencing to quantify six histone modifications, reduced representation bisulphite sequencing to quantify DNA methylation and mRNA microarrays to quantify gene expression before and after 7 days of chondrogenic differentiation of MSCs in an alginate scaffold. To add to the clinical relevance of our observations, the study is based on primary bone marrow-derived MSCs from four donors, allowing us to investigate inter-individual variations.

**Results:**

We see two levels of relationship between epigenetic marking and gene expression. First, a large number of genes ontogenetically linked to MSC properties and the musculoskeletal system are epigenetically prepatterned by moderate changes in H3K4me3 and H3K9ac near transcription start sites. Most of these genes remain transcriptionally unaltered. Second, transcriptionally upregulated genes, more closely associated with chondrogenesis, are marked by H3K36me3 in gene bodies, highly increased H3K4me3 and H3K9ac on promoters and 5' end of genes, and increased H3K27ac and H3K4me1 marking in at least one enhancer region per upregulated gene. Within the 7-day time frame, changes in promoter DNA methylation do not correlate significantly with changes in gene expression. Inter-donor variability analysis shows high level of similarity between the donors for this data set.

**Conclusions:**

Histone modifications, rather than DNA methylation, provide the primary epigenetic control of early differentiation of MSCs towards the chondrogenic lineage.

## Background

The best treatment of lesions of hyaline cartilage known to date is transplantation of *in vitro* expanded autologous chondrocytes [1]. However, as articular chondrocytes dedifferentiate during *in vitro* culture, the resulting tissue frequently consists of a mixture of hyaline and fibrous cartilage. A better strategy may be to produce an implant of hyaline cartilage by *in vitro* tissue engineering using mesenchymal stem cells (MSCs) and a biomaterial. Several approaches including a variety of scaffold systems have been used to induce chondrogenic differentiation of MSCs, but so far all have failed to produce perfect hyaline cartilage for clinical use [1,2]. To achieve this, it is important to understand the processes that regulate chondrogenic differentiation of MSCs.

Changes in epigenetic marks are known to be important regulatory factors in stem cell differentiation. Gene expression is correlated with the level of post-translational modifications of histone tails and DNA methylation of promoter regions. For embryonic stem cells (ESCs), lineage commitment is associated with repression of genes important for differentiation along other lineages [3-5]. This process frequently involves the polycomb-group mediated trimethylation of lysine 27 of histone 3 (H3K27me3) and DNA methylation of the promoter regions [6,7]. Transcriptionally active genes are marked by monoubiquitination of H2B followed by trimethylation of H3K4 (H3K4me3) and H3K9 acetylation (H3K9ac) on nucleosomes close to the transcription start site (TSS). Other histone modifications, including H3K36me3, are associated with transcriptional elongation and occur throughout the body of transcribed genes. Additional transcriptional control is provided by *cis*-acting regulatory regions marked by H3K4me1 and H3K27ac [4,5,8,9].

Less is known about the epigenetic control of gene expression during differentiation of multipotent stem cells. Analysis of genes regulating adipogenic differentiation of human MSCs revealed dynamic changes in histone marks reminiscent of those seen in ESCs [10] and modest changes in promoter DNA methylation [11]. Recent studies of differentiation of murine myogenic precursor lines and *in vivo* differentiation of murine hair follicle stem cells have focused on the importance of H3K27me3 [12,13]. Likewise, studies of human hematopoietic stem cells have focused on changes in genes concomitantly marked by H3K4me3 and H3K27me3 and changes in DNA methylation [6,14-16]. Except for a few studies of the DNA methylation status of some genes involved during *in vitro* chondrogenesis [17-19], nothing is known to date about epigenetic changes during chondrogenic differentiation of MSCs.

Embryological chondrogenesis is controlled by a complex interplay of growth- and transcription factors. Among the essential secreted factors are Tgfβ and Bmp proteins [20]. For *in vitro* differentiation of human MSCs a combination of TGFβ and BMP2 yields the most hyaline-like cartilage [21]. *In vivo*, the Sox9 transcription factor (TF) is essential through the entire process of chondrogenesis [22] but the co-activators L-Sox5 and Sox6 are also important for cartilage formation [23,24]. *In vitro*, transfection of SOX9, 5 and 6 together is sufficient to induce permanent cartilage in mesenchymal precursors [25]. Using TGFβ1 and BMP2 to induce chondrogenic differentiation in hMSCs in a self-gelling alginate scaffold system, we have recently shown that the differentiated cells upregulate the extracellular matrix (ECM) molecules typical of hyaline cartilage, and downregulate molecules typical of MSC functionality [26]. In this system, the SOX TFs are upregulated sufficiently to allow transcription of all essential ECM molecules. We reasoned that a system where availability of TFs is not rate limiting should be suitable to study the relationship between changes in gene expression and concomitant epigenetic changes. To this end we used whole-genome chromatin immunoprecipitation and deep sequencing (ChIP-seq) to quantify H3K4me3, H3K9ac, H327me3, H3K36me3, H3K4me1 and H3K27ac, and reduced representation bisulphite sequencing (RRBS) to quantify DNA methylation in hMSCs before and after 7 days of chondrogenic differentiation. To add to the clinical relevance of our observations, the study is based on primary bone marrow-derived MSCs (BM-MSC) from four donors, which allowed us to also investigate the inter-individual variations in the dynamics of epigenetic marks. We find evidence of two levels of relationship between epigenetic marking and gene expression during chondrogenic differentiation of MSCs: a prepatterning level marked by moderate changes in H3K4me3 and H3K9ac near TSSs and no or little change in gene expression, and a level associated with transcriptional upregulation marked by increased H3K36me3 along the gene body and highly increased H3K4me3 and H3K9ac. The first level is generally associated with the musculoskeletal system and MSC functionality, while the second level is more specifically associated with chondrogenesis. Within the 7-day time frame, changes in promoter DNA methylation do not correlate significantly with changes in gene expression.

## Results

### Chondrogenic differentiation of hBM-MSCs in 3D scaffold

**Figure 1:**
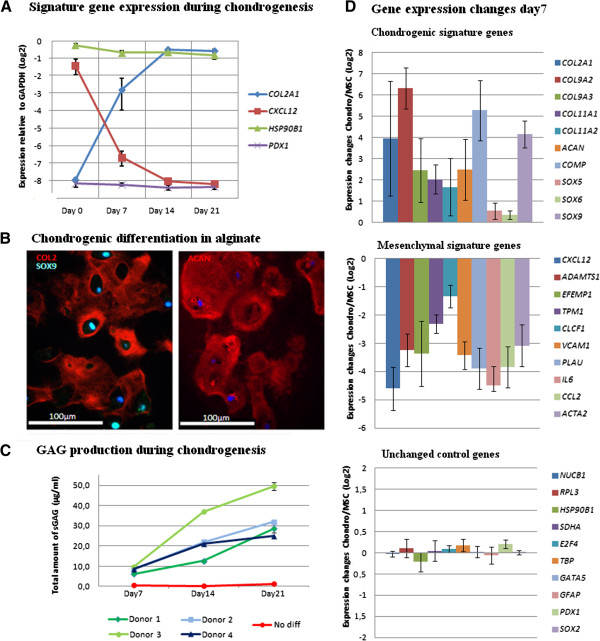
**Chondrogenic differentiation of hBM-MSCs in 3D scaffold. (A)** Levels of gene expression over 3 weeks of chondrogenic differentiation for chondrogenic and MSC signature genes and control genes measured by microarray analysis. Shown are mean values for donors 2–4. Error bars are standard deviations (SD). **(B)** Immunofluorescence histochemical stainings for SOX9 and type II collagen (left) and aggrecan (right) after three weeks of chondrogenic differentiation in one representative donor (Donor 2). **(C)** Secretion of sulfated glycosaminoglycans (sGAG) during three weeks of culture in alginate discs in either normal culture medium (No diff) or in the chondrogenic differentiation medium. Data presented are mean values of technical quadruplicates for each of the four donors. Error bars show standard deviations of the technical replicates. **(D)** Microarray expression analysis of chondrogenic and mesenchymal signature genes and unchanged control genes. Log2 transformed mean values for change in expression between day 0 and day 7 of chondrogenic differentiation are shown relative to GAPDH expression. Shown are mean values of the results for the four donors (mean of biological quadruplicates). Error bars are SD

In order to create an implant which resembles articular cartilage of the knee, we embedded MSCs in alginate discs with the thickness of knee cartilage and exposed these constructs to a differentiation cocktail containing TGFB1, BMP2 and dexamethasone [26]. Evidence of chondrogenic differentiation is shown by upregulation of the chondrocyte marker gene *COL2A1* and downregulation of the MSC marker gene *CXCL12* (Figure 1A), by the distribution of type II collagen and aggrecan throughout the extracellular space and SOX9 in the nuclei at three weeks of differentiation (Figure 1B), and by the increased synthesis of glycosaminoglycans (GAGs) during the differentiation period (Figure 1C). Details of gene expression and ECM composition using this model system are described in [26].

To first focus our epigenetic analyses on one set of genes typical of differentiated chondrocytes and another set typical of undifferentiated MSCs, we chose 10 canonical chondrogenic (*COL2A1, COL9A2, COL9A3, COL11A1, COL11A2, ACAN, COMP, SOX5, SOX6* and *SOX9)* and mesenchymal *(CXCL12, IL6, VCAM1, CCL2, PLAU, CLCF1, ADAMTS1, EFEMP1, ACTA2* and *TPM1)* signature genes (Figure 1D). The chondrogenic signature genes were selected because collagens II, IX and XI are the molecules required for synthesis of the hyaline cartilage collagen heterofibrils, which make up approximately 40% of the dry weight of hyaline cartilage. Aggrecan and COMP are two other essential components of hyaline ECM, while SOX9, 6 and 5 are essential transcription factors. The MSC signature genes were selected because they encode chemokines, cytokines and ECM degradation molecules important for the role played by MSCs in inflammation (*CXCL12, IL6, VCAM1, CCL2, PLAU, CLCF1, ADAMTS1),* or represent molecules involved in differentiation along other lineages *(ACTA2, TPM1)*. All these genes are widely distributed throughout the genome, suggesting that if genes respond similarly to the differentiation process, this impact occurs genome-wide. We also included ten control genes representing highly (*NUCB1, RPL3* and *HSP90B1)* and moderately *(E2F4, TBP, SDHA)* expressed housekeeping genes as well as non-expressed endodermal *(GATA5, PDX1)* and ectodermal *(SOX2, GFAP*) genes. The change in mRNA levels for these genes between day 0 and day 7 is shown in Figure 1D, and shows upregulation of the chondrogenic signature genes, downregulation of the MSC signature genes and no change for the control genes. Lists of all up- and downregulated genes in this differentiation system are found in Additional file 1 and Additional file 2.

### Histone modifications in promoters and gene bodies

**Figure 2:**
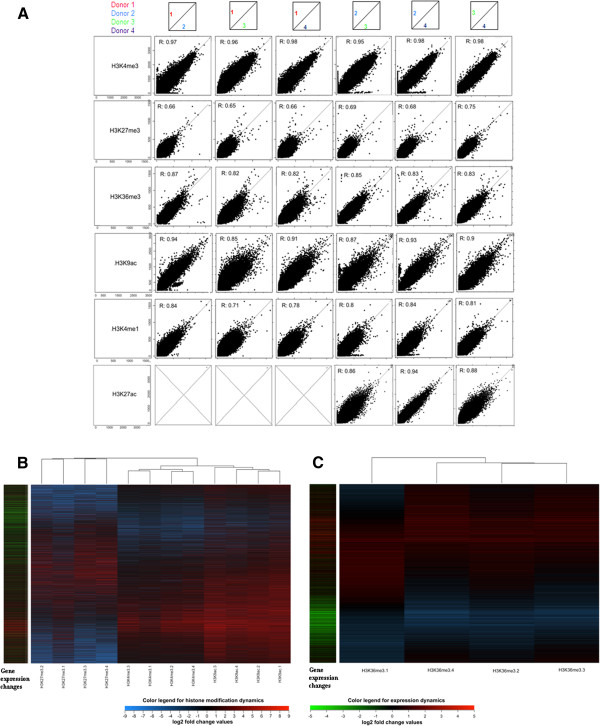
**(A) Correlation between all combinations of pairs of donors for enriched histone marks.** Each dot plot is a comparison between the level of enrichment in a given histone mark after 7 days of differentiation from two donors. The values in each plot are the number of reads observed in genome-wide 500 bp regions with enrichment of the histone mark in that particular region in cells from at least one of the two donors. Included within each dot plot is the Spearman's rho correlation coefficient. Results for H3K27ac for donor 1 are not available due to a failed experiment. **(B)** Heat-maps of changes in the course of 7 days of chondrogenic differentiation in promoter marks H3K27me3, H3K4me3 and H3K9ac. Rows represent 500 bp regions where at least one experiment produced at least 200 aligned reads. The columns show changes in the indicated histone modifications and are sorted using hierarchical clustering with the clusters represented at the top of the figure. The rows are sorted using Principal Component Analysis where the order of the rows is defined by the first two principal components. Colour bars at the bottom of the figure show the colour analogue to the log2 fold change in histone marking or gene expression. Each column has 96 013 rows

To describe the relationship between changes in gene expression and changes observed in the histone tails in nucleosomes, we quantified the presence of the transcriptionally permissive marks H3K4me3 and H3K9ac and the repressive mark H3K27me3 around the respective TSS in BM-MSCs from all four donors before and after 7 days of chondrogenic differentiation. We also quantified H3K36me3 throughout the length of the gene body. The genome-wide inter-donor variability of enrichment for the different histone marks during the differentiation period is presented in Figure 2A, and shows a remarkable similarity between donors for H3K4me3 (R ≥ 0.95), H3K9ac (R ≥ 0.85) and H3K36me3 (R ≥ 0.82). We found greater between-donor variability for H3K27me3 around the TSS, but this likely reflects the relatively low enrichment levels for this mark.

To extend the comparison to include both increases and decreases in histone marks, and to relate these changes to changes in gene expression, heat maps of genome-wide changes in facilitating promoter marks H3K4me3 and H3K9ac and restrictive promoter mark H4K27me3 are shown in Figure 2B, and a heat map of changes in H3K36me3 is shown in Figure 2C, in both cases directly comparable to changes in gene expression. As expected the maps representing the different promoter marks cluster together, with no apparent order between the donors. Upregulated H3K4me3 marks are seen to be closely related to promoters of genes with increased expression, while upregulated H3K9ac marks genes with increased expression, but also unchanged and perhaps even some downregulated genes. Upregulated H3K27me3 clusters with downregulated genes (Figure 2B). For H3K36me3 the relationship between enhanced histone mark and increased gene expression seems even stronger (Figure 2C).

**Figure 3:**
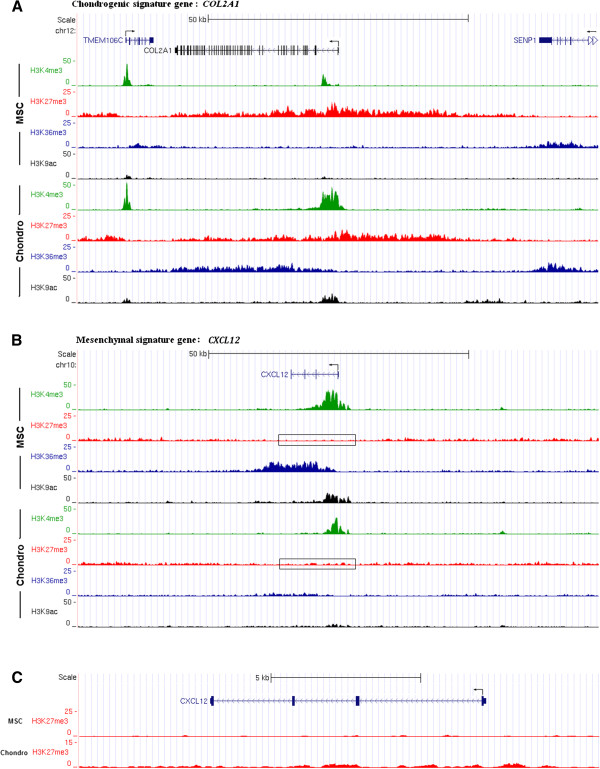
**Histone modification tracks of individual signature genes. (A)** ChIP-seq signal tracks for histone modifications in a 100 kb window around the TSS of chondrogenic marker gene *COL2A1*. Exon-intron structures and coding strand direction are depicted on top. UCSC Genome Browser on Human Feb. 2009 (GRCh37/hg19) assembly. **(B)** ChIP-seq signal tracks for histone modifications in a 100 kb window around the TSS of MSC marker gene *CXCL12*. **(C)** ChIP-seq signal tracks for H3K27me3 in a higher resolution of MSC marker gene *CXCL12*

**Figure 4:**
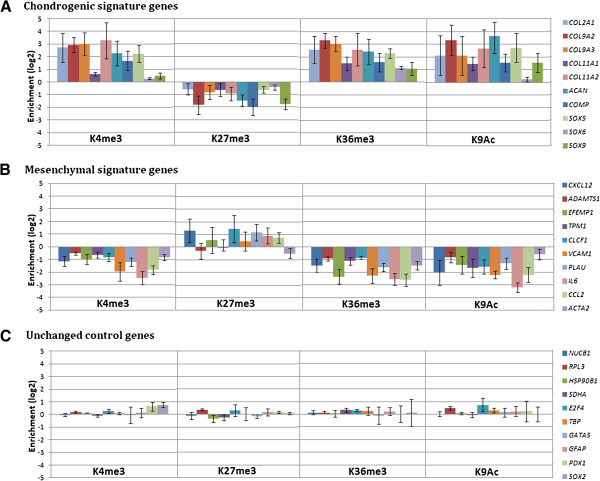
**Profiling of epigenetic changes near signature genes. (A)** Changes in histone marks for chondrogenic signature genes between day 0 and day 7 of chondrogenic differentiation. For H3K4me3, H3K27me3 and H3K9ac a 4 kb window around the TSS of all known transcripts was compared between the differentiation stages, while K36me3 reads were counted over the whole length of the longest known transcript. Shown is the log2 transformed histone enrichment ratio between differentiated cells and MSCs. Mean values of the results from the four donors (biological replicates) are shown. Error bars are SD. **(B)** Changes in histone marks for MSC signature genes between day 0 and day 7 of chondrogenic differentiation. Mean values of four donors with SD. **(C)** Changes in histone marks for unchanged control genes between day 0 and day 7 of chondrogenic differentiation. Mean values of four donors with SD

Next we examined changes in histone modifications near upregulated chondrogenic signature genes, downregulated MSC genes and unchanged control genes. *COL2A1* and *CXCL12* are shown as representatives for the signature gene clusters (Figure 3). In MSCs, the promoter region of *COL2A1* is marked by wide enrichment in H3K27me3 and no H3K4me3, which just shows a small peak in the first intron. This inactive state changed to a state marked by elevated H3K4me3 around the TSS. Interestingly, we did not observe a complete disappearance of H3K27me3. While this is reminiscent of the bivalent, or poised state described for non-transcribed, developmentally important genes in ESCs [4,5,8], we note that at this time there was already a high level of transcription of the *COL2A1* gene (Figure 1B). Thus, this apparent overlap is more likely to reflect that not all cells in the population express COL2A1 at this stage of differentiation. Promoter enrichment in H3K9ac and gene body occupancy by H3K36me3 are consistent with ongoing transcription (Figure 3A). In contrast, for the downregulated MSC marker gene *CXCL12* we note the disappearance of H3K9ac and H3K36me3 and a restriction in H3K4me3 occupancy to the vicinity of the TSS. H3K27me3 increases significantly around the TSS but remains low at day 7 (Figure 3B). The persistently non-transcribed gene *PDX1* shows no activity marks and high levels of H3K27me3 throughout the gene and extending on both sides (Additional file 3A). As expected, the highly expressed housekeeping gene *HSP90B1* displays high levels of the activity marks H3K4me3, H3K9ac and H3K36me3 and no H3K27me3 (Additional file 3B). Analysis of the set of chondrogenic signature genes reveals a >2-fold increase in H3K4me3 and H3K9ac for most of the genes and in H3K36me3 in all of the genes (Figure 4A). H3K27me3 shows a >2-fold decrease during differentiation for four of the chondrogenic signature genes. All the downregulated MSC genes show reduced H3K4me3, H3K36me3 and H3K9ac on day 7 of differentiation, while the genes are still being expressed at moderate levels. Most of these genes also show a minor increase in H3K27me3 (Figure 4B). Control genes do not show any significant changes in any of the investigated marks (Figure 4C). Thus, for practically all signature genes examined, there is a close association between gene expression and detection of marks of active genes, and an inverse relationship between mRNA level and H3K27me3.

### Genome wide correlation between changes in H3K4me3 and changes in gene expression

Based on the association observed between increased H3K4me3 and increased expression for the chondrogenic signature genes, we next investigated if increased H3K4me3 could be found for all genes that were transcriptionally upregulated. Of the 488 genes with increased mRNA levels, 90% were associated with increased H3K4me3 around the TSS, with approximately 65% showing a >2-fold increase (data not shown). We next determined if changes in H3K4me3 were restricted to genes with concomitant changes in mRNA levels. While approximately 1000 genes were significantly either up- or downregulated upon chondrogenic differentiation, >2600 genes showed significant changes in H3K4me3 near the TSS (Additional file 4A). Gene ontology (GO)-term analysis of genes with increased H3K4me3 showed enrichment of functions linked to skeletal system development, ECM and chondrocyte differentiation. The functional terms for the genes with decreasing H3K4me3 were more general and associated with regulation of cytoskeleton, apoptosis, cell development or metabolic processes (Additional file 4B). Genome-wide, only 25% of the genes associated with a >2-fold change in H3K4me3 showed significant change in expression on day 7 (p<0.05) (Additional file 5A). Hardly any of these genes would change in expression over the next two weeks of differentiation, as the vast majority of genes that show change in expression during the first three weeks do so during the first week in this differentiation system. These results indicate that H3K4me3 enrichment occurs during chondrogenic differentiation on genes that are ontologically close to cartilage and musculoskeletal systems, but that these genes are not transcriptionally activated at the ≥2-fold level defined as significant in this study.

**Figure 5:**
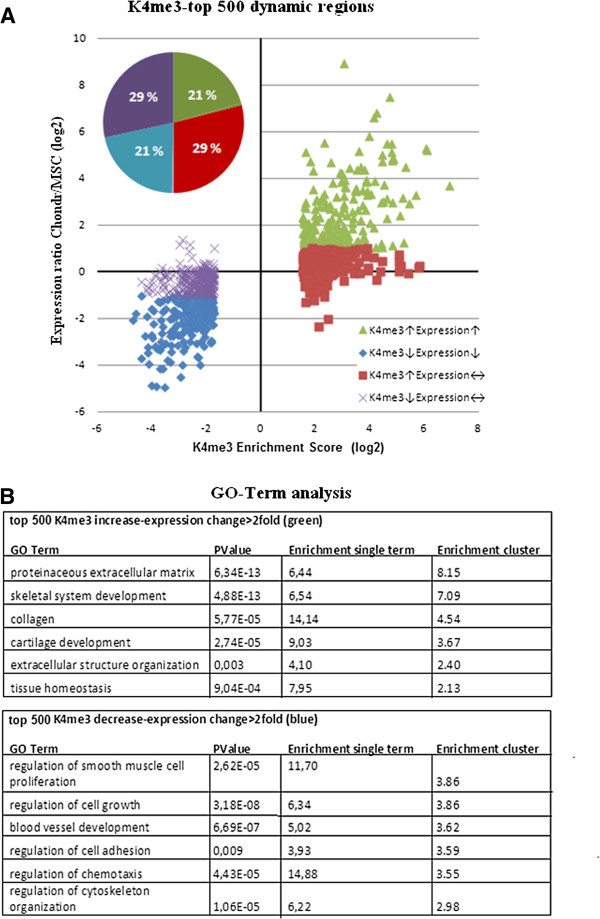
**Correlation between changes in gene expression and changes in promoter mark H3K4me3. (A)** Association between change in gene expression and change in H3K4me3 among the 500 regions which showed the highest increase or decrease in H3K4me3 in promoter regions. **(B)** GO term analysis of genes within the top 500 H3K4me3 clusters which reached >2-fold expression change at day 7 of chondrogenic differentiation. DAVID cluster analysis was performed and one typical term per cluster is shown

To determine if the magnitude of change in H3K4me3 might be a predictor for a change in gene expression we looked at the 500 regions with the greatest increase or decrease in H3K4me3 (Figure 5A). We found that 41% of the genes with the greatest increase, and 42% of the genes with the greatest decrease in H3K4me3 showed >2-fold transcriptional up- or downregulation, respectively (Additional file 4C), compared with 25% for all genes with changes in H3K4me3. GO-term analysis of the upregulated genes showed a closer correlation to ECM and cartilage (Figure 5B), while the downregulated genes showed properties associated with MSCs: differentiation to other lineages and association with angiogenesis and inflammation (Figure 5B). If the selection was limited further to include only the 100 TSS regions with the greatest level of up- or downregulation in H3K4me3, 65% of the associated genes were found to be differentially expressed (data not shown). Of the genes associated with the greatest upregulation of H3K4me3, but with <2-fold increase in mRNA level, many were either significantly upregulated (p<0.05) but <2-fold increased, were upregulated at a later time point during differentiation [26], showed activation of alternative promoters or were wrongly annotated (Additional file 6). In fact, only 8 of the 100 genes with the highest increase in H3K4me3 were transcriptionally unaffected. A similar conclusion could be drawn from the analysis of the genes that were most reduced in H3K4me3. Thus, the greatest changes in H3K4me3 were almost always associated with a change in gene expression.

### Correlation between combination of histone modifications and gene expression

Most of the genes that were transcriptionally unchanged but with highly increased H3K4me3 had no increase in either H3K9ac or H3K36me3 or both. This suggests that lack of H3K9ac and/or H3K36me3 occupancy could explain why transcription of these genes did not change despite their increased H3K4me3, or that changes in H3K9ac and H3K36me3 did not occur because transcription did not change despite changes in H3K4me3 marking. Based on this observation, we determined the relationship between increased gene expression and the presence of all the gene-associated histone modifications, alone or in combination (Additional file 5). Not surprisingly, H3K4me3 and H3K9ac were co-regulated around a large number of genes. However, the histone mark that most closely correlated with increased gene expression was H3K36me3. Of the 488 genes with increased mRNA levels, 94% had the H3K36me3 modification, 85% of these were numerically increased from day 0 to day 7 of differentiation, while 26% were >2-fold increased (Additional file 5 and data not shown). Taken together, this shows that highly increased levels of H3K4me3 and H3K9ac and upregulated levels of H3K36me3 are found near practically every transcriptionally upregulated gene in this differentiation system.

### The impact of cis-acting regulatory elements

**Figure 6:**
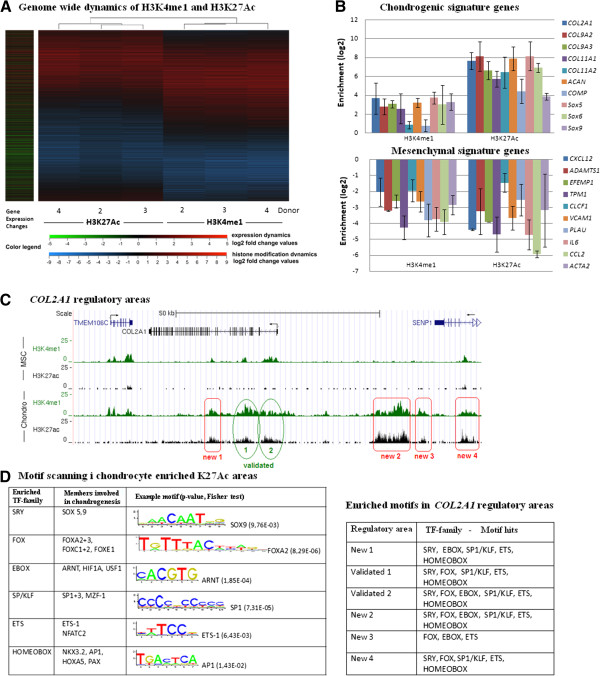
**Finding potential cis-regulatory elements. (A)** Heat maps of H3K4me1 and H3K27ac marks in regions located within 50 kb of the 5^′^ end of genes with significant changes in expression in the course of 7 days of chondrogenic differentiation in donors 2**–**4. Data for donor 1 are not available due to a failed experiment. Definition of rows, sorting of rows and clustering of columns as described in the Legend to Figure 2B. Each column has 262 533 rows. **(B)** Profiles of the regulatory histone modification marks for selected chondrogenic and mesenchymal signature genes. Shown is the 500 bp bin with the highest histone enrichment ratio for the indicated genes in a 50 kb area as mean value of three donors. Error bars are SD. **(C)** ChIP-seq signal tracks for H3K4me1 and H3K27ac in a 50 kb area on either side of the TSS of the *COL2A1* gene. Validated (green) and potential new [29] regulatory areas are highlighted. **(D)** Left side: Motif scanning for transcription factor binding sites in regions enriched for H3K27ac in differentiated cells. Example motif shown from Jaspar database. The members of the TF families are TFs known to be involved in chondrogenesis which share similar binding motifs. Right side: Motif hits in regulatory areas around the *COL2A1* gene

Regulation of gene transcription is controlled by factors acting in promoter regions as well as distant *cis*-regulatory elements. Different histone marks have been described to correlate with promoter and enhancer regions. Active enhancers are known to be marked by H3K4me1 and H3K27ac [27,28]. Heat maps for the associations between genes with changes in expression and H3K4me1 and H3K27ac marking within 50 kb of the TSS of these genes is shown in Figure 6A. A very clear association is seen between up- or down-regulation in gene expression and corresponding up- or down-regulation of the histone marks. Genome-wide, more than 70% of the upregulated genes were in the vicinity of upregulated enhancer regions, but since the number of regions with increased H3K4me1 or H3K27ac was high, only approximately 15% of the genes within 50 kb of changes in these marks showed changes in mRNA expression (Additional file 7). This suggests that enhancer regions are important for most upregulated genes, but that the predictive power of a change in regulatory regions for the transcription of nearby genes is limited. A genome-wide list of genes which change in expression and show increase or decrease in one of these two regulatory marks in a 50 kb area is given as Additional file 8 (H3K4me1) and Additional file 9 (H3K27ac).

In order to identify potential chondrogenic enhancer regions, we investigated changes in these regulatory marks within 50 kb of the TSS of genes that changed transcriptionally during chondrogenic differentiation. For our signature genes, we found at least one site of >2 fold enrichment in both histone modifications near every chondrogenic signature gene, and at least one site with a significant >2 fold decrease of these marks near every MSC signature gene (Figure 6B). None of the unchanged control genes showed changes in H3K4me1 or H3K27ac (data not shown). The changes in H3K4me1 and H3K27ac near the chondrogenic marker gene *COL2A1* are shown in Figure 6C. We detected six areas with enrichment in both H3K4me1 and H3K27ac in the chondrogenically differentiated cells. Two of these could be identified as described and validated enhancer regions with binding sites for SOX9 [30-33]. The other four regions (Figure 6C, marked in red) have not yet been described, and are likely to be new regulatory elements of importance for the regulation of *COL2A1* expression.

### Transcription factor binding sites in cis-acting regulatory regions - Motif search

The impact of changes in *cis*-acting regulatory elements on the regulation of gene expression most likely occurs through the binding of TFs. In order to identify TFs which may be involved in the regulation of chondrogenesis we performed a motif search in all areas with increase of H3K27ac during the differentiation process, using JASPAR, Transfac and Uniprobe databases. We found enrichment in motifs which could bind TFs belonging to several families (Figure 6D). The SRY family is especially important in the context of chondrogenesis, as it contains SOX5, 6 and 9, which are essential and sufficient to start the differentiation process [23,34]. The literature also contains evidence of involvement in chondrogenesis of members of all the other families with enriched binding motifs in these regions [35-38]. Interestingly, for most of these TF families binding motifs could be found also within the new regulatory areas close to the *COL2A1* gene, further supporting their potential role in chondrogenic differentiation of MSCs (Figure 6D). Most significantly, new regions 1, 2 and 4 could be shown to contain 3, 8 and 2 sequences identical to the SOX9 binding motif (AACAAT), respectively, while new region 3 contains no such sequences.

### Donor variability

**Figure 7:**
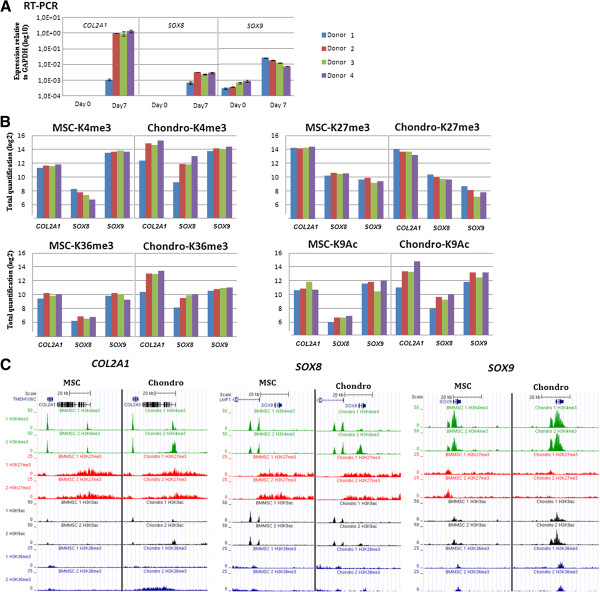
**Donor variability for single genes. (A)** Gene expression analysis by real-time RT-qPCR. *COL2A1*, *SOX8* and *SOX9* are shown relative to *GAPDH* expression for the individual donors. Note log10 scale. Each sample was measured in triplicate. Mean values shown, error bars reflect the Standard deviation. **(B)** Changes in histone modifications for individual donors. For H3K4me3, H3K27me3 and H3K9ac the sum of aligned reads within a 4 kb window around the TSS of all Ensembl transcripts of indicated genes is shown in both differentiation stages, while for H3K36me3 the sum of the reads for the longest transcript for each ensembl gene is given. **(C)** ChIP-seq signal tracks for donor 1 and 2 showing the indicated histone modifications in a 60 kb window around the TSS of indicated genes

While the data comparing histone modifications genome-wide between all four donors shows a low level of overall donor variability (Figure 2), some individual genes show variation between donors in mRNA levels and associated histone modifications. One example is *COL2A1,* where the gene expression on day 7 for donor 1 was considerably lower than for the other three donors. A similar observation was made for the upregulated gene *SOX8*, but not for *SOX9* (Figure 7A). When changes in histone modifications were examined for these genes, a much lower rate of change was observed for donor 1 for H3K4me3, K36me3 and H3K9ac for *COL2A1* and *SOX8*, but not for *SOX9* (Figure 7B and 7C), suggesting a correlation between gene expression and histone modification also at the single gene and individual donor level. Of the other genes identified which showed delayed transcriptional increase in donor 1 compared to donors 2–4, the majority was found to have decreased upregulation of H3K4me3, K36me3 and H3K9ac for donor 1 compared with donors 2–4. Interestingly, there were no clear differences in these histone marks, H3K27me3 or DNA methylation at *COL2A1* or *SOX8* in the undifferentiated cells, which suggests that these marks are not sufficient to predict the delayed differentiation response of donor 1.

### DNA methylation

To determine if there is a relationship between DNA methylation and changes in gene expression before and after 7 days of chondrogenic differentiation, genome-wide DNA methylation in CpG-rich regions was mapped using RRBS. More than 2 × 10^6^ unique CpGs were observed with at least 10X coverage in most samples. The overall methylation patterns were highly similar between samples and followed expected patterns. CpGs in CpG-rich regions (including CpG islands) tended to be hypomethylated (<10% median methylation across covered CpGs) while CpGs in CpG-poor regions tended to be hypermethylated (>50% median methylation; Additional file 10A). This relationship was preserved when the promoter regions were examined specifically. A highly significant correlation was found between the average methylation values for the MSC and the average methylation values for the chondrocytes in promoter regions (r^2^ = 0.78), with the methylation states of the vast majority of promoters remaining unchanged [11]. For the MSC and chondrocyte signature genes, promoters were hypomethylated both before and after chondrogenesis, which suggests that DNA methylation is unlikely to play a major role in regulating these key genes during chondrogenesis. Genome-wide, only 37 promoters (0.14% of those examined), including those of 9 microRNAs, showed a mean increase of at least 50% in methylation across the donors, and only 284 (1.1%) showed more than a 25% increase. However, except for *GAS6* (growth-arrest specific 6, five-fold downregulated, increased promoter methylation), these genes were generally expressed at low levels both before and after differentiation and no correlation to changes in the investigated histone marks could be detected. Moreover, at least 15 of the 37 genes that gained >50% methylation across the donors were already hypermethylated in one of the donors prior to differentiation, which suggests that the observed promoter methylation events are not likely to be directly linked to regulation of chondrogenesis.

## Discussion

### Chondrogenic differentiation opens up a new epigenetic landscape in MSCs

*In vitro* tissue engineering of hyaline cartilage implants using MSCs and a biomaterial may well represent the best future treatment option for patients with lesions of articular cartilage. To be applicable to a wide range of patients the tissue engineering strategy should be robust, with little between-donor variability. Preferably, the differentiation process should be induced synchronously in all the cells used. A priori, this may be difficult to achieve: MSCs from different donors behave differently in cell expansion and differentiation cultures, and MSCs are known to be heterogenous both phenotypically and functionally [39,40]. In contrast, using the alginate disc differentiation procedure described here, hyaline ECM molecules are produced by the vast majority of cells in all donors tested so far [26] (Küchler AM and Brinchmann JE, unpublished results). However, there is a delay between upregulation of mRNA and detection of ECM molecules, and ECM molecule synthesis does not seem to be synchronous. Against this background, we observe a remarkable similarity between the histone modifications induced after seven days of chondrogenic differentiation using bone marrow-derived MSCs from four donors. At this time, a new epigenetic landscape is opened up in these cells. Changes in the H3K4me3 and H3K9ac modifications occur near the TSS in thousands of genes, many of which are ontogenetically linked to skeletal system development, ECM, chondrocyte differentiation and MSC functionality. Although these modifications are known to be associated with initiation of transcription [4,41], no significant change in mRNA levels is actually observed in the majority of these genes. With the level of change in gene expression set as significant in this study (≥2-fold) it is possible that a small change in gene expression may accompany these changes in histone marks, or perhaps a greater change in a very small subset of cells. However, these changes are most reminiscent of the prepatterning of developmental gene expression by H3K4me3 recently observed in zebrafish before zygotic genome activation [42]. It is possible that every chondrogenic differentiation protocol would induce an epigenetic state that maintains activation potential of genes required both for the desired and also for related differentiation pathways [15]. It is also possible that certain components of the present differentiation cocktail, e.g. BMP2, may improve chondrogenic differentiation (Jakobsen RB, Østrup E, Mikkelsen TS and Brinchmann JE, unpublished observations) while at the same time setting an epigenetic stage also for bone differentiation.

### Close correlation between histone marks and gene expression

Although many genes within the general domain of the musculoskeletal system show increased H3K4me3 following 7 days of chondrogenic differentiation, only the genes with the highest increase in H3K4me3 could be correlated with increased gene expression. The distribution of H3K4me3 and H3K9ac was, in many instances, very similar, suggesting co-occurrence on the same histone tails. H3K4me3 and H3K9ac may poise genes for transcription without transcription actually occurring. However, increased H3K4me3 and H3K9ac in promoter regions is, on its own, unlikely to explain the close correlation between highly increased H3K4me3 and H3K9ac and increased transcriptional activity. Increase in the distribution of these modifications within the gene body may conceivably explain the increased rate of transcription, as H3K4me3 has also been suggested to stimulate transcriptional elongation [43]. However, the modification most closely correlated with increased transcription is H3K36me3. The combination of H3K4me3 with H3K36me3 is observed at practically every gene with increased mRNA level, and at few other genes. The following model may reconcile our results with current knowledge about the relationship between histone modifications and transcription. Trimethylation of H3K4 is regulated by several factors, including trithorax group proteins and cyclin-dependent kinase-9 (CDK9) of the positive transcription elongation factor-b complex (P-TEFb) [43,44]. The regulation of H3K9ac is not well known, but its co-occurrence with H3K4me3 has been described [7,45], and the two modifications may have common chromatin readers [46,47]. These modifications frequently co-occur with RNA polymerase II [45]. However, transcriptional elongation does not occur in the absence of trimethylated H3K36 along the gene body. Methylation of H3K36 is performed by the methyltransferase Set2 and is, like H3K4 trimethylation, dependent on CDK9 [44,48]. CDK9 and Set2 are part of a large complex of proteins which is involved in the release of paused polymerase II [41]. With the factors responsible for trimethylation of H3K36 being part of the transcription elongation complex, it is uncertain at this time whether H3K36me3 is a prerequisite for, or a consequence of the passage of this complex along the gene body. In either case transcriptional elongation, heralded by H3K36me3, is the most specific correlate of actively transcribed genes in the present data set. Further research will, hopefully, explain how transcriptional elongation is restricted to a set of genes with particular relevance for hyaline chondrogenesis in this differentiation model.

### Apparent co-occurrence of active and repressive marks

Genes regulated both by H3K4me3 and H3K27me3 in the promoter region are called bivalent genes. ESCs are known to have a high proportion of bivalent genes, particularly genes responsible for specification along lineages [4,5,8]. These genes are not transcribed as long as differentiation is inhibited. On a cell population basis, we also observed what looks like bivalent genes, both among the upregulated chondrogenic signature genes and among the downregulated MSC signature genes. On day 7 of differentiation, transcription of all these genes was observed. We cannot rule out the possibility that there are genes among these that are actively transcribed in the presence of both H3K4me3 and H3K27me3 near the TSS. More likely, however, is an explanation based on non-synchronous changes in histone modifications and gene transcription between the different cells in a heterogeneous population. This would imply, for *COL2A1*, for instance, that one subset of MSCs lose H3K27me3 and gain H3K4me3 (and H3K36me3) at day 7. This subset is responsible for the observed increased level of *COL2A1* mRNA, while another subset with retained H3K27me3 near the *COL2A1* promoter does not support transcription. The level of *COL2A1* mRNA increases further through days 14 and 21, which is consistent with subsequent loss of H3K27me3 and gain of active marks in cells that are still repressed at day 7.

### Relationship between distal cis-regulatory elements and gene expression

The genome-wide analysis of all 50 kb areas around TSS revealed a correlation between gene expression and changes in the regulatory histone marks H3K4me1 or H3K27ac. For the signature genes, every upregulated chondrogenic gene is associated with at least one upregulated enhancer region and every downregulated MSC-specific gene is associated with at least one downregulated enhancer region, whereas none of the control genes show any such change within the 50 kb region. For the *COL2A1* marker gene, 4 new cis-regulatory regions were discovered, all containing binding motifs for TFs essential for chondrogenesis. This strongly suggests that distal cis-regulatory regions impact importantly on the regulation of gene expression in this differentiation model. However, most of the histone modifications marking cis-regulatory regions occurred >50 kb away from genes with changed expression, suggesting that the enhancers may be far removed from the target gene.

## Conclusion

We have performed genome-wide quantitative epigenetic analysis on biological replicates of primary human MSCs undergoing chondrogenic differentiation in alginate disc scaffolds relevant for tissue engineering of hyaline cartilage. The differentiation cocktail and 3D culture induce changes in H3K4me3 and H3K9ac in genes associated with the musculoskeletal system, but mostly in the absence of changes in gene expression. Increased gene expression may be predicted by particularly high increase in H3K4me3 and by increase in H3K36me3. This implicates transcriptional elongation as the specificity conferring, rate limiting step in the differentiation process. *Cis*-regulatory activity marked by increasing H3K27ac and H3K4me1 is involved in transcriptional regulation in the vast majority of genes with altered expression. Within the 7-day time frame, changes in promoter DNA methylation do not correlate significantly with changes in gene expression.

## Methods

### Culture and validation of MSCs

The study was approved by the Regional Committee for Medical Research Ethics, Southern Norway. Bone marrow was obtained from four healthy voluntary donors following informed, written consent. The MSCs were isolated and cultured as described in [26]. The cells were validated as MSCs by expression of CD105, CD73, CD90 and CD44, and failure to express CD34, HLA- DR, CD45, CD14 and CD19. The multipotency of the MSCs was validated by differentiation to osteogenic and adipogenic lineages as previously described in [26].

### In vitro chondrogenic differentiation

The chondrogenic differentiation system was described previously [26]. Briefly, MSCs were established in a self-gelling system containing PRONOVA-LVG alginate and calcium-alginate-particles to a final alginate concentration of 1% and a final cell concentration of 5 × 10^6^ cells/ml. Chondrogenic differentiation was induced by 500 ng/ml BMP2, 10 ng/ml TGFB1 and 0.1 μM dexamethasone in serum free medium containing supplements as described [26].

### Validation of extracellular matrix synthesis

Fluorescence immunohistochemistry was performed as described [26] with reagents as specified in Additional file 11. The supernatants from disc cultures were analyzed for sulfated glycosaminoglycan content in technical quadruplicates for each donor using the Blyscan™ kit (Biocolor Ltd).

### RNA preparation and expression analysis

Total RNA was prepared using TRIzol (Invitrogen). Depolymerisation of the alginate discs to obtain single cells for RNA isolation was performed by enzymatic digestion using G-Lyase (kindly provided by professor Gudmund Skjåk-Bræk) as described in [26]. RT-qPCR was performed using TaqMan arrays (Applied Biosystems) and normalized to *GAPDH* expression. Results are presented as mean plus standard deviation of technical triplicates. Microarray assays were performed in uniplicates per donor and differentiation state using the Human-6 v3 Expression Beadchips (Illumina), and the analysis was performed using J-express 2009 [49].

### ChIP-seq

After degelling, cells were fixed in 1% formaldehyd for 10 min at 37°C and stored at −80°C. ChIP and Illumina sequencing library construction was performed as described in [3,4].

### Sequence analysis

Tracks were uploaded in a UCSC Genome Browser, using unique reads extended to 200 bp. Experiments were normalized by enforcing equal read counts. 500 bp bins were generated, and the coverage in each bin was calculated for both chip and control. For each bin, a fold change value and a poisson p-value was generated. P-values were FDR corrected. The read counts from all experiments were quantile normalized. Bins with a log2 fold change >0.2 or <−0.2 in at least three of the four replicates were defined as having an increased or decreased signal, respectively. For quantification of H3K27me3, H3K4me3, and H3K9ac the sum of aligned reads within 2 kb on both sides of the TSS for each gene was calculated. This was performed for all biological replicates for both MSCs and differentiated cells, and a mean for each cell population was calculated. For H3K36me3, with known functionality throughout the entire gene [50], the longest transcript for each Ensembl gene was selected. The total number of aligned reads within this transcript was then calculated for all biological replicates in both cell populations, and a mean for each population was calculated. For the profiling of H3K4me1 and H3K27ac signals the highest ratio of bins associated to a single gene was calculated.

#### Gene association

For promoter marks, Ensembl transcripts were used to identify transcripts with a 5^′^ end within 2 kb of bins. The transcript with the closest 5^′^ end was selected. For enhancer marks the association was made to the most differentially expressed gene within 50 kb. Microarray expression data were integrated using refseq IDs.

#### Motif enrichment analysis

For each histone mark, the top 5000 increasing and decreasing bins were selected. Bins next to each other were merged. The regions were scanned with motifs from Jaspar, Transfac and Uniprobe with a score threshold of 80%. Fisher test was used to compare the number of sequences with at least one hit and the number of sequences with no hits between the two sets. Wilcox test was used to compare the rank of total number of hits between the two sets. Motifs with a p-value < 0.05 from both tests were defined as significant.

### DNA methylation

RRBS was performed as previously described [51]. To analyze potential changes in promoter DNA methylation during chondrogenesis we examined 26,003 distinct TSSs annotated on the UCSC Genome Browser (derived from the gh19 refFlat database). For each sample, we assigned a methylation value to each TSS for which we had at least 5 distinct CpGs with at least 5X coverage each within −1,000 bp to +1,500 bp relative to the TSS. The methylation value for each such TSS was taken to be the median methylation value of all of the CpGs within this region with at least 5X coverage, using at least three of the four donors in each state.

### Sequences and data access

All ChIP-Seq and RRBS data have been deposited in public databases and can be accessed from the NIH Roadmap Project on Epigenomics website: http://www.roadmapepigenomics.org/ and in the NCBI GEO database under accession GSE19465.

## Abbreviations

MSC: Mesenchymal stem cell; BM-MSC: Human bone marrow-derived mesenchymal stem cells; ESC: Embryonal stem cell; ECM: Extracellular matrix; TSS: Transcriptional start site; TF: Transcription factor; ChIP-seq: Chromatin immunoprecipitation and deep sequencing; RRBS: Reduced representation bisulphite sequencing; GAG: Glycosaminoglycan; CDK9: Cyclin-dependent kinase-9; P-TEFb: Positive transcription elongation factor-b complex.

## Competing interest

The authors declare that none of them have competing interest of any kind.

## Authors’ contribution

SRH, TSM and JEB conceived of the study and its design, SRH and TH performed cell work and library preparation, JCB performed the biostatistical analyses, SRH, JCB, LAMZ, PC, TSM and JEB analysed the data, LW, RI, XZ, MJC, PB and HG made the library and performed the sequencing, SRH and JEB wrote the manuscript with substantial input from JCB, TH, PC and TSM and JEB supervised the study. All authors contributed to and approved the final manuscript for publication.

## Authors’ information

Reprint author: Jan E. Brinchmann MD, PhD

Institute of Immunology, Oslo University Hospital Rikshospitalet, PO Box 4950 Nydalen, 0424 Oslo, Norway

Telephone: +47 22 84 04 89

Fax: +47 22 85 10 58

E-mail: jan.brinchmann@rr-research.no

## Supplementary Material

Additional file 1 **Genes with up-regulated expression during chondrogenic differentiation.** Shown is the microarray expression data for all four donors, with mean values for chondrogenic and mesenchymal cells and the enrichment ratio as log2 transformed values. Click here for file

Additional file 2 **Genes with down-regulated expression during chondrogenic differentiation.** Shown is the microarray expression data for all four donors, with mean values for chondrogenic and mesenchymal cells and the enrichment ratio as log2 transformed values. Click here for file

Additional file 3 **Histone modification tracks of non-changed control genes.** ChIP-seq signal tracks for histone modifications in a 100 kb window around the TSS of non-expressed control gene *PDX1*. Exon-intron structures and coding strand direction are depicted on top. UCSC Genome Browser on Human Feb. 2009 (GRCh37/hg19) assembly. (A)ChIP-seq signal tracks for histone modifications in a 100 kb window around the TSS of highly expressed control gene *HSP90B1*. Click here for file

Additional file 4 **Correlation between changes in gene expression and changes in promoter mark H3K4me3. (A)** Genome-wide association between changes in gene expression (>2-fold, p<0.05) and changes in H3K4me3 (>2-fold in at least 3 out of 4 donors) during 7 days of differentiation. **(B)** GO term analysis of all genes where H3K4me3 changed >2-fold in 3 out of 4 donors. A cluster analysis using DAVID was performed, and one typical term per cluster is shown. **(C)** Counts of genes in the different expression clusters for all dynamic H3K4me3 regions and the 500 regions which changed the most in H3K4me3 (up or down) during one week of chondrogenesis. **(D)** GO term analysis for genes within the top 500 regions which increased or decreased in H3K4me3, but failed to reach a >2-fold expression change. DAVID analysis was performed, and one typical term per cluster is shown. Click here for file

Additional file 5 **Correlation between presence or absence of gene expression and change in promoter associated histone modifications. (A)** Correlation between changes in histone marks and presence or absence of changes in gene expression. Shown is the percentage of genes clustered according to gene expression within groups characterized by single or combined significant changes in histone modifications. Expression cluster: ↑: >2fold upregulated, ↓: >2fold downregulated, ↔: <2fold changed. Numbers are mean values for all four donors. **(B)** Table showing the total number of genes associated to the indicated dynamic regions and expression clusters. Numbers are mean values of all four donors. Click here for file

Additional file 6 **Analysis of genes within the Top 500 with increased H3K4me3 which did not meet significance criteriae for increased gene expression. (A)** Categories of genes which failed to reach the significance criteriae (>2-fold increase, p<0.05) for increased gene expression. **(B)** Details of gene expression analysis [52] and epigenetic profile (bottom) for *SOX5* as an example of a chondrogenic signature gene with <2-fold, but significantly changed mRNA expression. **(C)** ChIP-seq signal tracks for histone modifications of the *SOX5* gene. Exon-intron structures and coding strand direction are depicted on top. UCSC Genome Browser. **(D)** ChIP-seq signal tracks of *KLHL5* as an example for genes within the top 500 genes with increased H3K4me3 due to activation of an alternative promoter, without change in the level of mRNA expression. **(E)** Example of wrong gene annotation. ChIP-seq signal tracks for histone modifications and data for changes in gene expression for *RAB4B* and *MIA*. Click here for file

Additional file 7 **Correlation between gene expression and changes in regulatory histone marks. (A)** Correlation between changes in histone marks and presence or absence of changes in gene expression. Shown is the percentage of genes clustered according to gene expression within groups characterized by single cis-regulatory histone marks, Expression cluster: ↑: >2fold upregulated, ↓: >2fold downregulated, ↔: <2fold changed. **(B)** Statistics of correlation between changes in histone marks and gene expression. Shown is the percentage of genes in different expression clusters associated to the indicated >2fold changed histone modifications and their indicated combination. Table showing the total number of genes associated to the indicated dynamic regions and expression clusters. Numbers are mean values of all four donors. Click here for file

Additional file 8 **Genes which have a change in expression combined with a change in the level of H3K4me1 with in 50 kb of the TSS.** Shown is gene identification and localization with expression values followed by the values for H3K4me1 marking where this is significantly changed in nearby 500 bp bins. In addition the gene expression for the associated genes is given as mean values for the three investigated donors. Click here for file

Additional file 9 **Genes which have a change in expression combined with a change in the level of H3K27Ac with in 50 kb of the TSS.** Shown is gene identification and localization with expression values followed by the values for H3K27Ac marking where this is significantly changed in nearby 500 bp bins. In addition the gene expression for the associated genes is given as mean values for the three investigated donors. Click here for file

Additional file 10 **DNA-Methylation levels during chondrogenesis. (A)** Distribution of methylation levels for individual CpGs with at least 10X coverage in each sample, as a function of local CpG density (**+/−** 50 bp). Shown is data of one typical donor. **(B)** Comparison of the average MSC and Chondro methylation value for 13,210 distinct transcription start sites (TSSs) annotated on the UCSC Genome Browser. Click here for file

Additional file 11 Primers used in real-time RT-PCR and antibodies used in immunohistochemistry. Click here for file
