# Supplementary figures and images for: Genome-wide map of quantified epigenetic changes during in vitro chondrogenic differentiation of primary human mesenchymal stem cells
Source: BMC Genomics. 2013 Feb 15;14:105. doi: 10.1186/1471-2164-14-105 (PMC3620534; doi:10.1186/1471-2164-14-105)

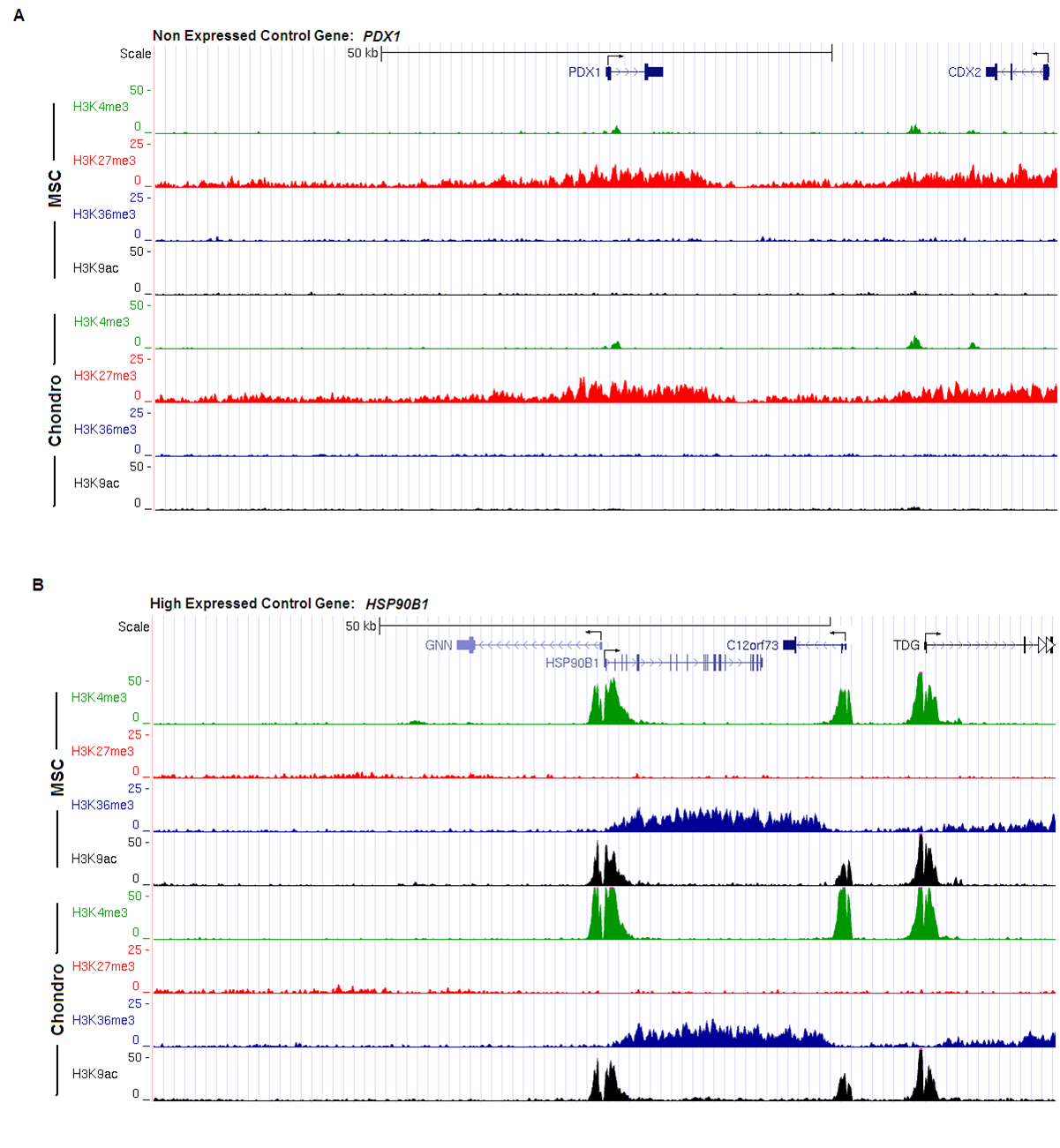

Supplement: Additional file 3 — Histone modification tracks of non-changed control genes. ChIP-seq signal tracks for histone modifications in a 100 kb window around the TSS of non-expressed control gene PDX1. Exon-intron structures and coding strand direction are depicted on top. UCSC Genome Browser on Human Feb. 2009 (GRCh37/hg19) assembly. (A)ChIP-seq signal tracks for histone modifications in a 100 kb window around the TSS of highly expressed control gene HSP90B1. [file 1471-2164-14-105-S3.tiff]

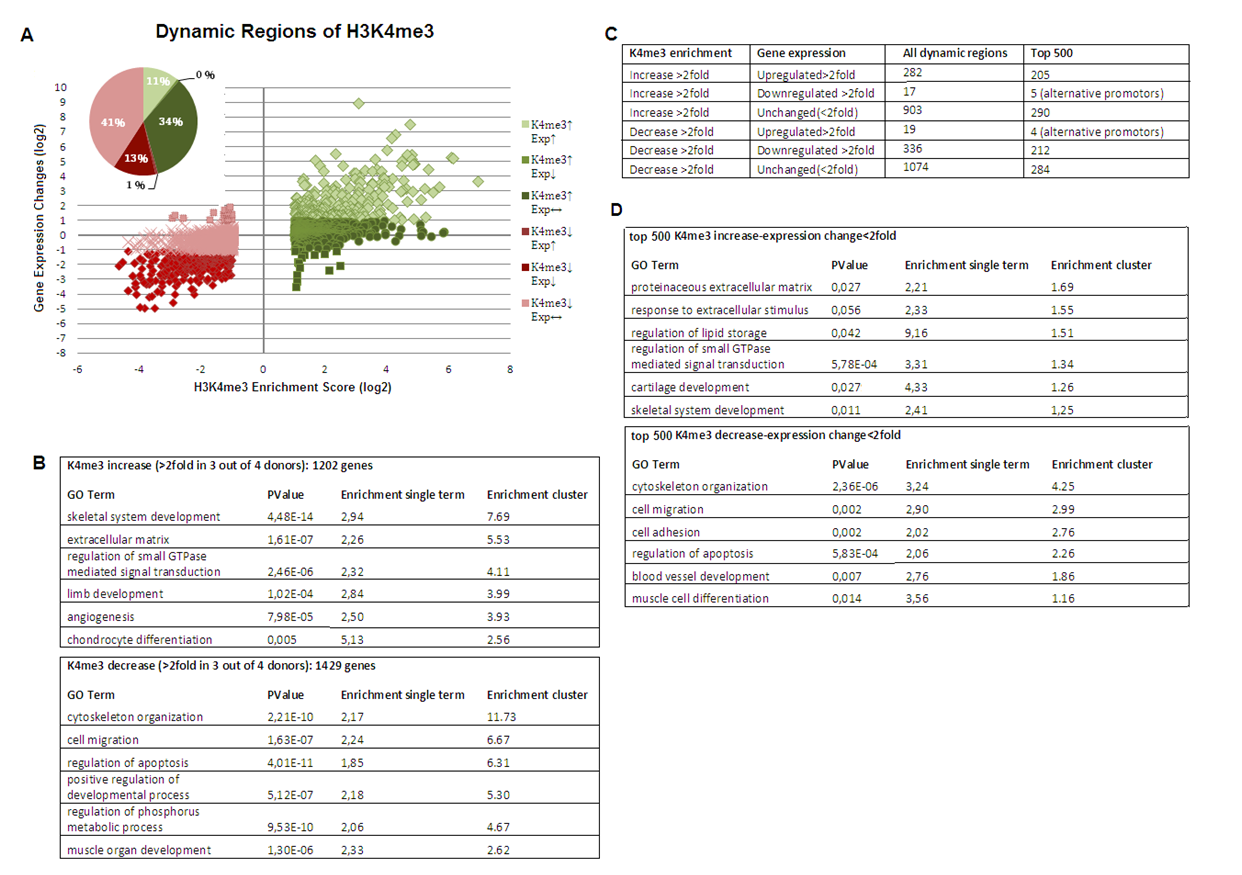

Supplement: Additional file 4 — Correlation between changes in gene expression and changes in promoter mark H3K4me3. (A) Genome-wide association between changes in gene expression (>2-fold, p<0.05) and changes in H3K4me3 (>2-fold in at least 3 out of 4 donors) during 7 days of differentiation. (B) GO term analysis of all genes where H3K4me3 changed >2-fold in 3 out of 4 donors. A cluster analysis using DAVID was performed, and one typical term per cluster is shown. (C) Counts of genes in the different expression clusters for all dynamic H3K4me3 regions and the 500 regions which changed the most in H3K4me3 (up or down) during one week of chondrogenesis. (D) GO term analysis for genes within the top 500 regions which increased or decreased in H3K4me3, but failed to reach a >2-fold expression change. DAVID analysis was performed, and one typical term per cluster is shown. [file 1471-2164-14-105-S4.tiff]

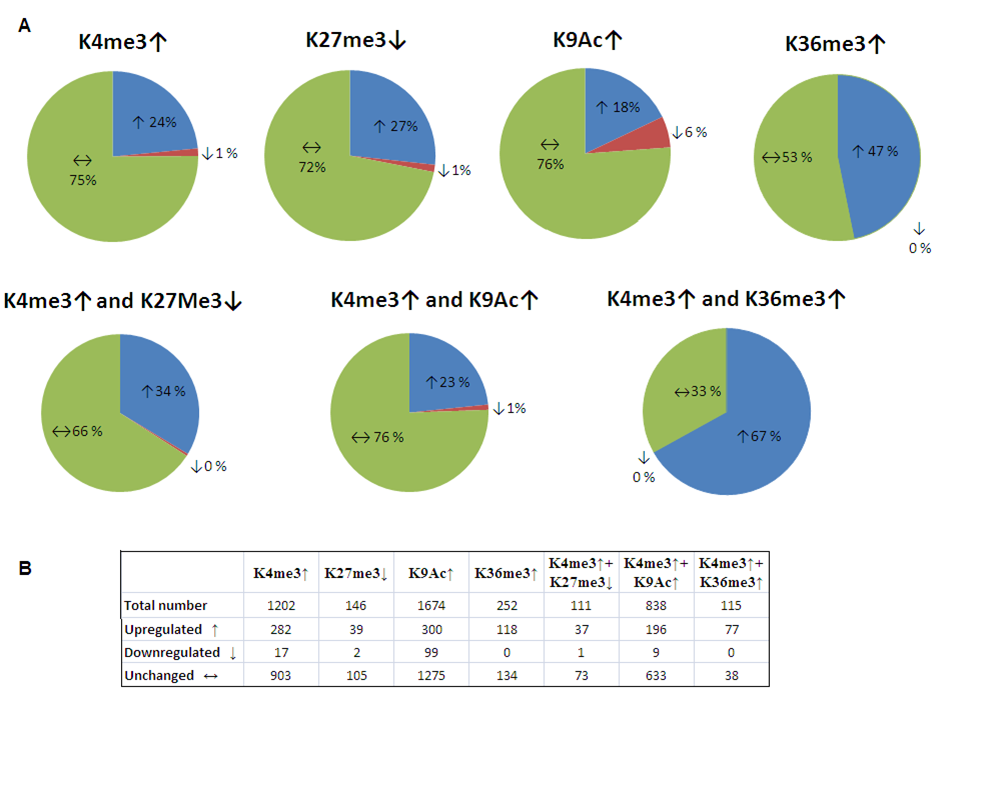

Supplement: Additional file 5 — Correlation between presence or absence of gene expression and change in promoter associated histone modifications. (A) Correlation between changes in histone marks and presence or absence of changes in gene expression. Shown is the percentage of genes clustered according to gene expression within groups characterized by single or combined significant changes in histone modifications. Expression cluster: ↑: >2fold upregulated, ↓: >2fold downregulated, ↔: <2fold changed. Numbers are mean values for all four donors. (B) Table showing the total number of genes associated to the indicated dynamic regions and expression clusters. Numbers are mean values of all four donors. [file 1471-2164-14-105-S5.tiff]

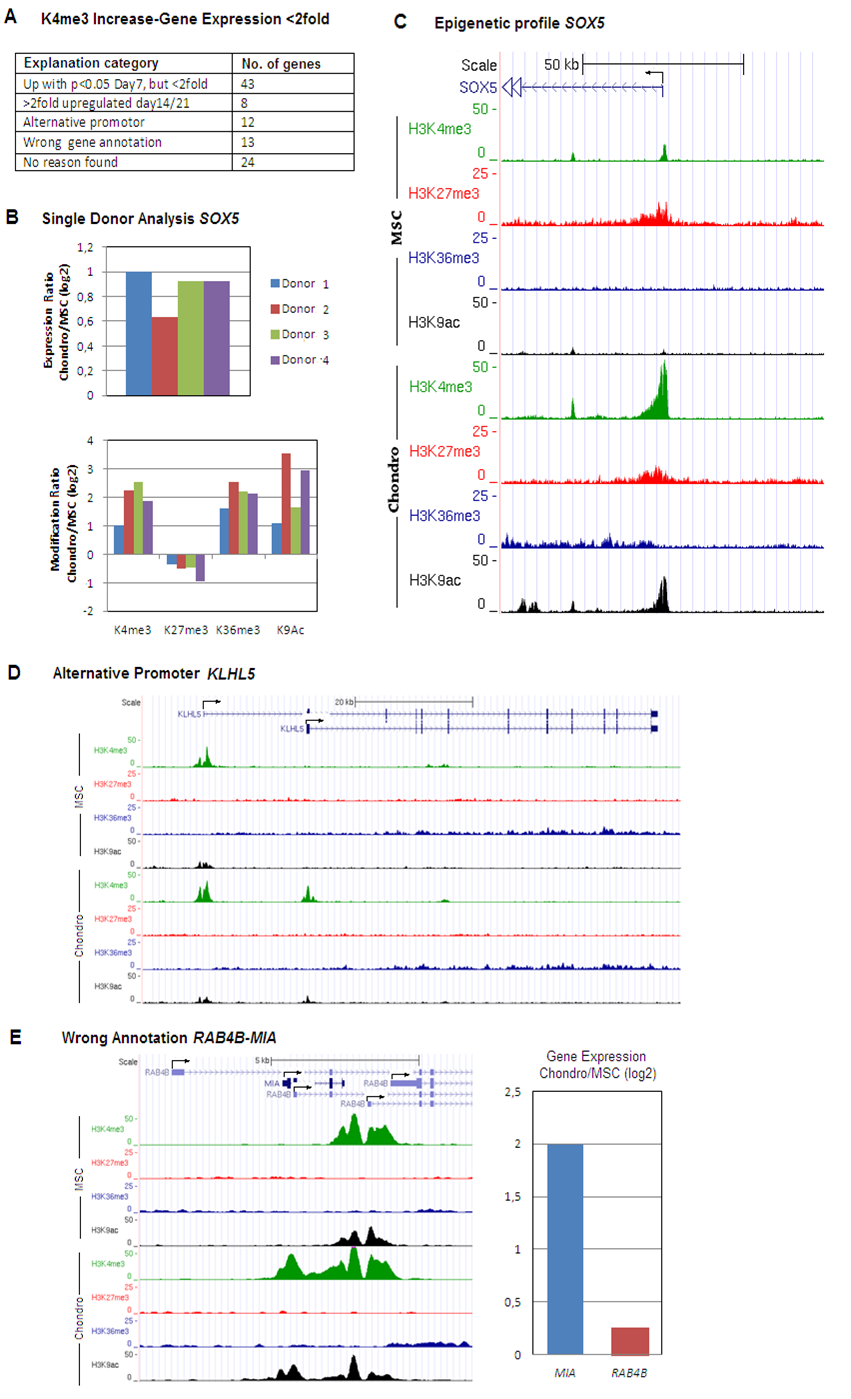

Supplement: Additional file 6 — Analysis of genes within the Top 500 with increased H3K4me3 which did not meet significance criteriae for increased gene expression. (A) Categories of genes which failed to reach the significance criteriae (>2-fold increase, p<0.05) for increased gene expression. (B) Details of gene expression analysis [52] and epigenetic profile (bottom) for SOX5 as an example of a chondrogenic signature gene with <2-fold, but significantly changed mRNA expression. (C) ChIP-seq signal tracks for histone modifications of the SOX5 gene. Exon-intron structures and coding strand direction are depicted on top. UCSC Genome Browser. (D) ChIP-seq signal tracks of KLHL5 as an example for genes within the top 500 genes with increased H3K4me3 due to activation of an alternative promoter, without change in the level of mRNA expression. (E) Example of wrong gene annotation. ChIP-seq signal tracks for histone modifications and data for changes in gene expression for RAB4B and MIA. [file 1471-2164-14-105-S6.tiff]

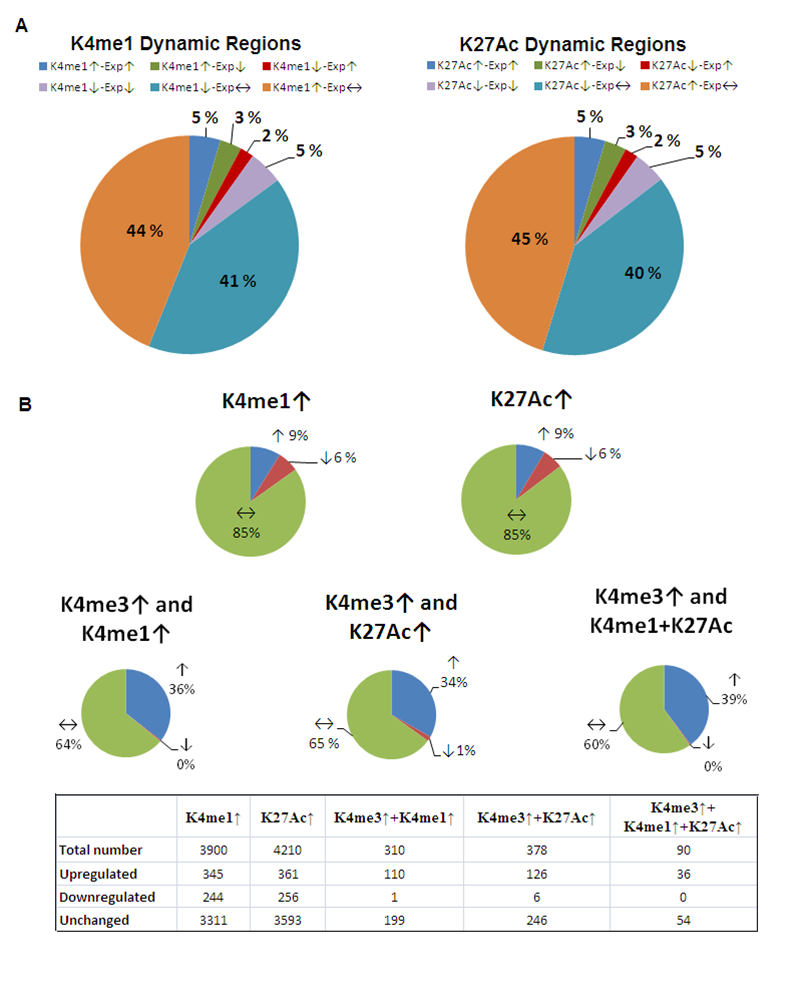

Supplement: Additional file 7 — Correlation between gene expression and changes in regulatory histone marks. (A) Correlation between changes in histone marks and presence or absence of changes in gene expression. Shown is the percentage of genes clustered according to gene expression within groups characterized by single cis-regulatory histone marks, Expression cluster: ↑: >2fold upregulated, ↓: >2fold downregulated, ↔: <2fold changed. (B) Statistics of correlation between changes in histone marks and gene expression. Shown is the percentage of genes in different expression clusters associated to the indicated >2fold changed histone modifications and their indicated combination. Table showing the total number of genes associated to the indicated dynamic regions and expression clusters. Numbers are mean values of all four donors. [file 1471-2164-14-105-S7.tiff]

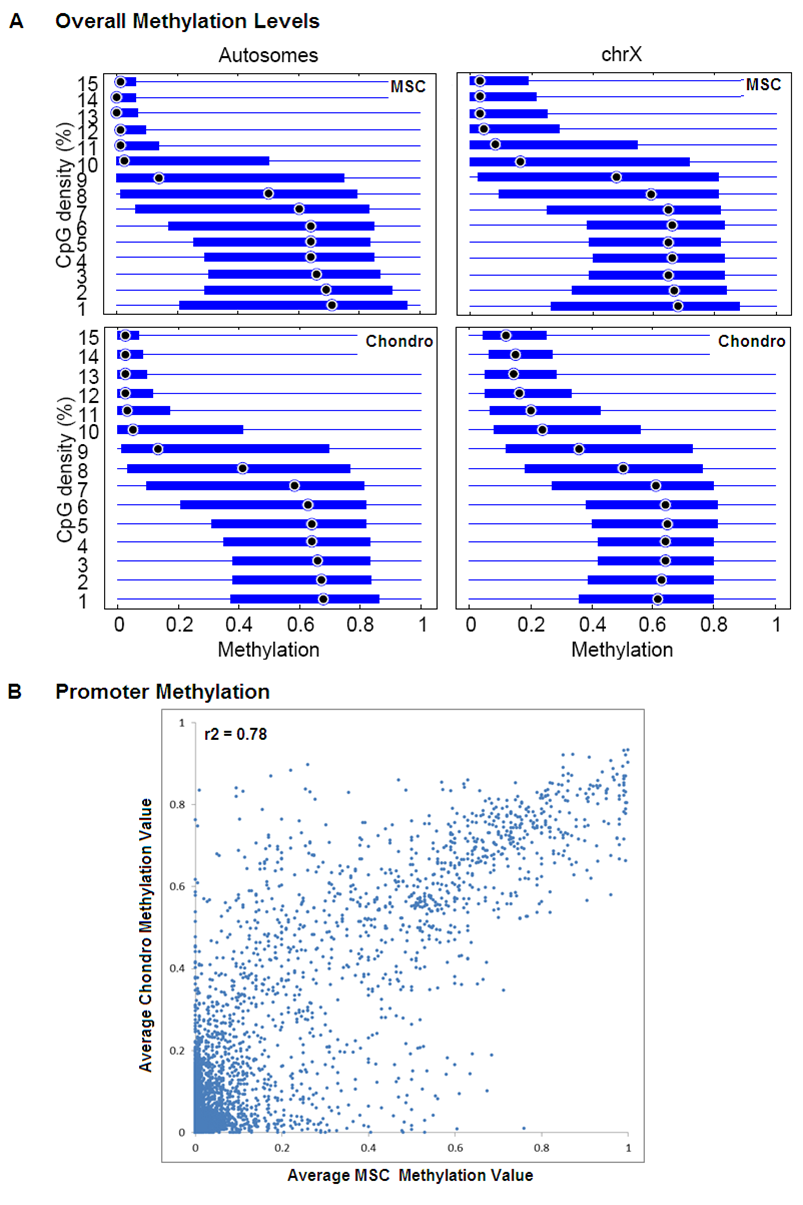

Supplement: Additional file 10 — DNA-Methylation levels during chondrogenesis. (A) Distribution of methylation levels for individual CpGs with at least 10X coverage in each sample, as a function of local CpG density (+/− 50 bp). Shown is data of one typical donor. (B) Comparison of the average MSC and Chondro methylation value for 13,210 distinct transcription start sites (TSSs) annotated on the UCSC Genome Browser. [file 1471-2164-14-105-S10.tiff]
