# Supplementary material for: Genome-wide map of quantified epigenetic changes during in vitro chondrogenic differentiation of primary human mesenchymal stem cells
Source: BMC Genomics. 2013 Feb 15;14:105. doi: 10.1186/1471-2164-14-105 (PMC3620534; doi:10.1186/1471-2164-14-105)
Supplement: Additional file 11 — Primers used in real-time RT-PCR and antibodies used in immunohistochemistry. [file 1471-2164-14-105-S11.docx]

## Additional file 7: Primers used in real-time RT-PCR and antibodies used in immunohistochemistry

| **Protein** | **Gene symbol** | **Primer for RT-PCR (Taqman assay no., Applied biosystems)** | **Antibodies for immunohistochemistry** | | |
| --- | --- | --- | --- | --- | --- |
|  |  |  | **Designation (concentration)** | **Specification** | **Company** |
| Type II collagen | COL2A1 | Hs00264051_m1 | II-4C11 (0.83μg/ml) | Mouse IgG1 | MP Biomedicals |
| SRY -box containing gene 9 | SOX9 | Hs00165814_m1 | AB5535 (0.2 μg/ml) | Rabbit | Millipore |
| SRY -box containing gene 8 | SOX8 | Hs00232723_m1 | --- | --- | --- |
| Aggrecan | ACAN | --- | 969D4D11 (4.55μg/ml) | Mouse IgG1 | BioSource |
